# A primitive type of renin-expressing lymphocyte protects the organism against infections

**DOI:** 10.1038/s41598-021-86629-w

**Published:** 2021-03-31

**Authors:** Brian C. Belyea, Araceli E. Santiago, Wilson A. Vasconez, Vidya K. Nagalakshmi, Fang Xu, Theodore C. Mehalic, Maria Luisa S. Sequeira-Lopez, R. Ariel Gomez

**Affiliations:** Department of Pediatrics, Child Health Research Center, University of Virginia School of Medicine, Charlottesville, VA USA

**Keywords:** Gene regulation in immune cells, Innate immune cells

## Abstract

The hormone renin plays a crucial role in the regulation of blood pressure and fluid-electrolyte homeostasis. Normally, renin is synthesized by juxtaglomerular (JG) cells, a specialized group of myoepithelial cells located near the entrance to the kidney glomeruli. In response to low blood pressure and/or a decrease in extracellular fluid volume (as it occurs during dehydration, hypotension, or septic shock) JG cells respond by releasing renin to the circulation to reestablish homeostasis. Interestingly, renin-expressing cells also exist outside of the kidney, where their function has remained a mystery. We discovered a unique type of renin-expressing B-1 lymphocyte that may have unrecognized roles in defending the organism against infections. These cells synthesize renin, entrap and phagocyte bacteria and control bacterial growth. The ability of renin-bearing lymphocytes to control infections—which is enhanced by the presence of renin—adds a novel, previously unsuspected dimension to the defense role of renin-expressing cells, linking the endocrine control of circulatory homeostasis with the immune control of infections to ensure survival.

## Introduction

Renin-expressing cells emerged in nature over 400 million years ago^1,2^. Throughout evolution, they have acquired numerous defensive functions that rendered them as perfect machines to ensure our survival in response to a variety of homeostatic threats^3^. They control blood pressure, fluid-electrolyte balance, vascular development, glomerular regeneration and may participate in the regulation of oxygen delivery to tissues^1,3^. Although renin cells were first discovered in the juxtaglomerular areas of the adult kidney arterioles, from where they release renin to the circulation to regulate blood pressure and fluid-electrolyte homeostasis, their appearance in this organ is a late event in their developmental history^1^. In fact, during early mouse and human development, cells that express renin emerge in multiple tissues and organs before they appear in the kidney^1^. The function of renin cells outside the kidney has remained a mystery and the subject of great speculation. We report here the discovery of a primitive type of renin-expressing cell within hematopoietic organs that persists throughout adulthood and may have hitherto unrecognized roles in defending the organism against infections. These cells possess unique capabilities to trap and phagocyte bacteria and control bacterial growth. The ability of renin-bearing lymphocytes to control infections adds a novel and unsuspected dimension to the defense role of renin-expressing cells.

## Results

**Figure 1:**
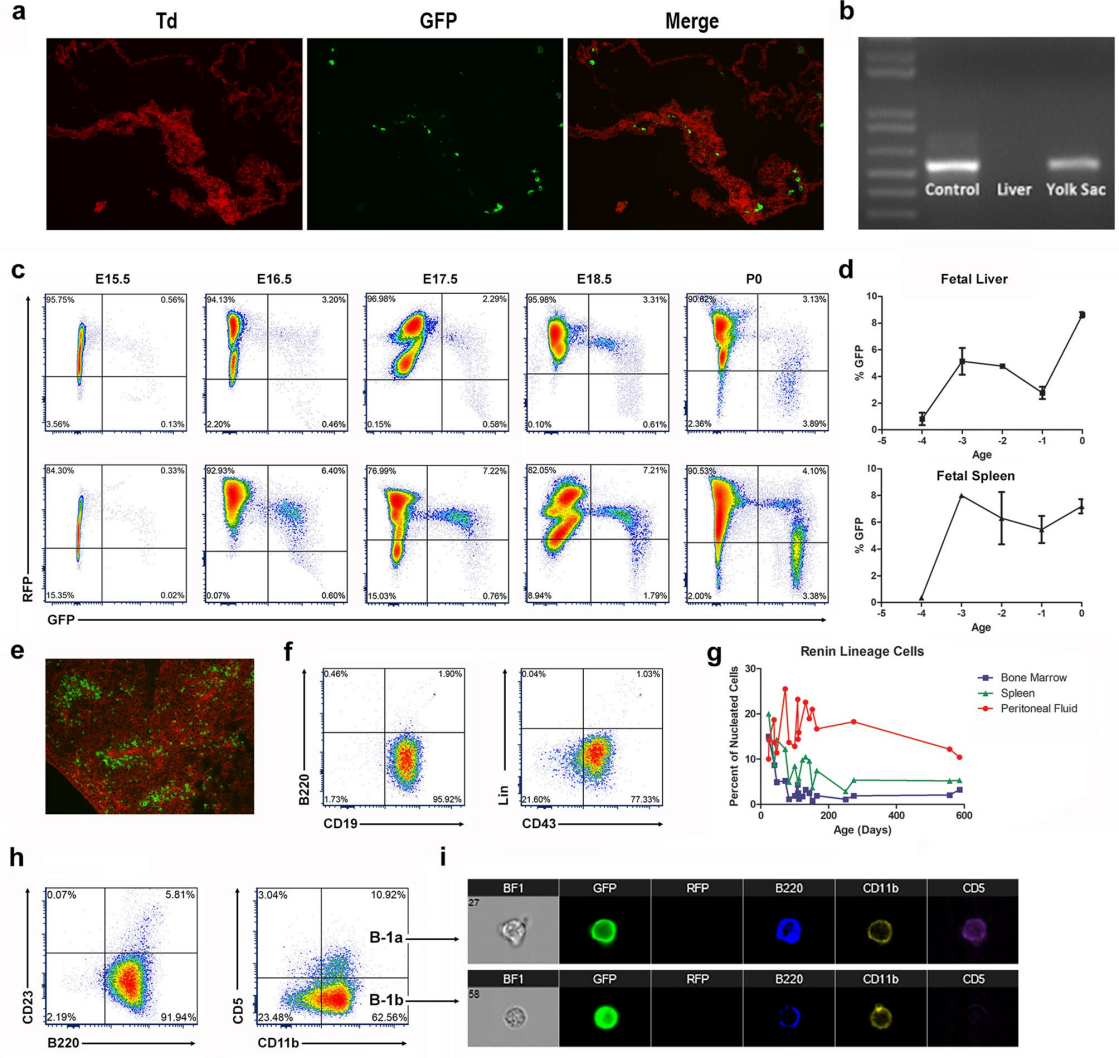
Renin-expressing cells arise during mid-gestation as B-1 progenitor cells and persist throughout adult life in the peritoneal cavity as B-1 B cells. (**a**) Renin progenitors appear in the yolk sac at E11.5 as individual green fluorescent protein positive (GFP^+^) cells within the yolk sac tissue of *Ren1*^*dcre/*+^*;mTmG* mice. (**b**) Semi-quantitative RT-PCR confirmed renin expression in the yolk sac at E11.5. Newborn kidneys and E11.5 livers were used as positive and negative controls respectively. (**c**) Flow cytometry representative plots show that renin-lineage cells (GFP^+^, x-axis) appear in the fetal liver and spleen at E15.5. With advancing gestational age (E15.5–P0, left to right), there is an increase in the percentage of GFP^+^ cells in both the fetal liver (top panel) and fetal spleen (bottom panel) (Here, GFP represents cells that have expressed renin and red fluorescent protein (RFP) represents cells that have not expressed renin). (**d**) Percentage of renin progenitors from E15.5 (− 4) to P0 using flow cytometry. (**e**) Renin lineage (GFP^+^) cells are present in the spleen within the white pulp in newborn mice. (**f**) Renin-expressing cells in the fetal tissues show a B-1 progenitor phenotype (CD19^+^, B220^dim^, CD43^+^, and Lin^−^). (**g**) The percentage of renin-lineage cells decreases in the bone marrow and spleen over time but persists in the peritoneal cavity throughout adult life. (**h**) Renin lineage (GFP^+^) cells in the peritoneal cavity are B-1b B cells (B220^+^, CD23^−^, CD11b^+^, CD5^−^) and B-1a B cells (B220^+^, CD23^−^, CD11b^+^, CD5^+^). (**i**) IMAGESTREAM flow cytometry shows that renin-lineage (GFP^+^) peritoneal cells express B220, CD11b, and + /− CD5. Cells that express CD5 are B-1a B cells, and cells that do not express CD5 are B-1b B cells.

Using lineage-tracing, flow cytometry, fluorescence imaging, and gene expression analysis, we examined the temporal appearance, distribution, identity, and evolution of renin progenitors throughout hematopoietic ontogeny. Renin-expressing, hematopoietic progenitors first appear within the yolk sac during mid-gestation. Using reporter mice (*Ren1*^*dcre/*+^*; mTmG*), which express green fluorescent protein (GFP) in all cells that expressed Ren1 and their descendants^4–6^, we identified rare GFP^+^ / renin^+^ cells first within the yolk sac at E11.5 (Fig. 1a,b). Their presence within this organ is transient and not found beyond E13.5. These dual hematopoietic and renin-bearing precursors appear later in the fetal liver and spleen at ~ E15.5 (Fig. 1c). As embryonic development progresses, the cells increase in number within the fetal liver and spleen, representing approximately 10% of hematopoietic cells within these tissues at the time of birth (Fig. 1d,e). To define the identity of these cells, we performed immunophenotyping using flow cytometry and a panel of well-characterized cell surface antibodies (Supplementary Table S1)^7–9^. In the fetal liver and spleen, the renin lineage cells express B lymphocyte cell surface markers CD19 and CD43, however they have dim B220 expression and are negative for a cocktail of lineage markers (“Lin”), consistent with a B-1 progenitor immunophenotype (B220^dim^CD19^+^CD43^+^Lin^−^)^10^ (Fig. 1f).

Following birth, renin lineage cells are found throughout the hematopoietic system, including bone marrow, spleen, and peripheral blood. Because B-1 cells have been observed in serosal compartments^7,11^, we confirmed that the mouse peritoneal cavity harbored renin-expressing cells with a lymphocyte pedigree (Fig. 1g). Whereas the proportion of renin cells in the peritoneal cavity is maintained throughout adult life, their proportion in the bone marrow and spleen diminishes with age (Fig. 1g). Renin lineage (GFP^+^) cells from the bone marrow, spleen, and peripheral blood are B-2 B lymphocytes (B220^+^CD19^+^CD23^+/−^CD11b^−^)^4^. However, renin progenitors in the peritoneal cavity are B-1 B cells (B220^dim^CD23^−^CD11b^+^CD5^+/−^) (Fig. 1h,i).

To confirm that renin-expressing progenitors in the fetal hematopoietic tissues give rise to mature lymphocytes in the adult, we performed transplant studies. We isolated hematopoietic cells from the fetal livers of E16.5 *Ren1*^*dcre/*+^*;mTmG* reporter mice and injected these cells into the tail vein of irradiated wildtype adult mice. Transplant of fetal liver cells gave rise to GFP^+^ B cells within the bone marrow, spleen, peripheral blood, and peritoneal cavity of recipient mice. Indeed, the percentage of GFP^+^ cells in the bone marrow and spleen of transplant recipient mice was similar to adult reporter mice (Supplementary Fig. S1a,b). The phenotype of the renin-lineage cells within the transplant recipients was also similar to that of the age-matched reporter mice (Supplementary Fig. S1a,b). Thus, these transplant studies confirm that renin-expressing hematopoietic progenitors during fetal life give rise to B-1 and B-2 cells in adult animals.

Until recently, the origin of B-1 lymphocytes has been unclear^12^. Our studies indicate that B-1 lymphocytes which had expressed renin during early development, originate within the yolk sac during the initial wave of primitive B lymphopoiesis and then expand within the fetal liver and spleen prior to the development of definitive hematopoiesis. Those cells persist during adult life as B-1 B cells in the peritoneal cavity and, to a lesser extent, as B-2 B cells in the bone marrow, spleen, and peripheral blood. The percent of B-1 B cells in the peritoneal cavity which are from the renin lineage is 25–45% depending on age (data not shown).

**Figure 2:**
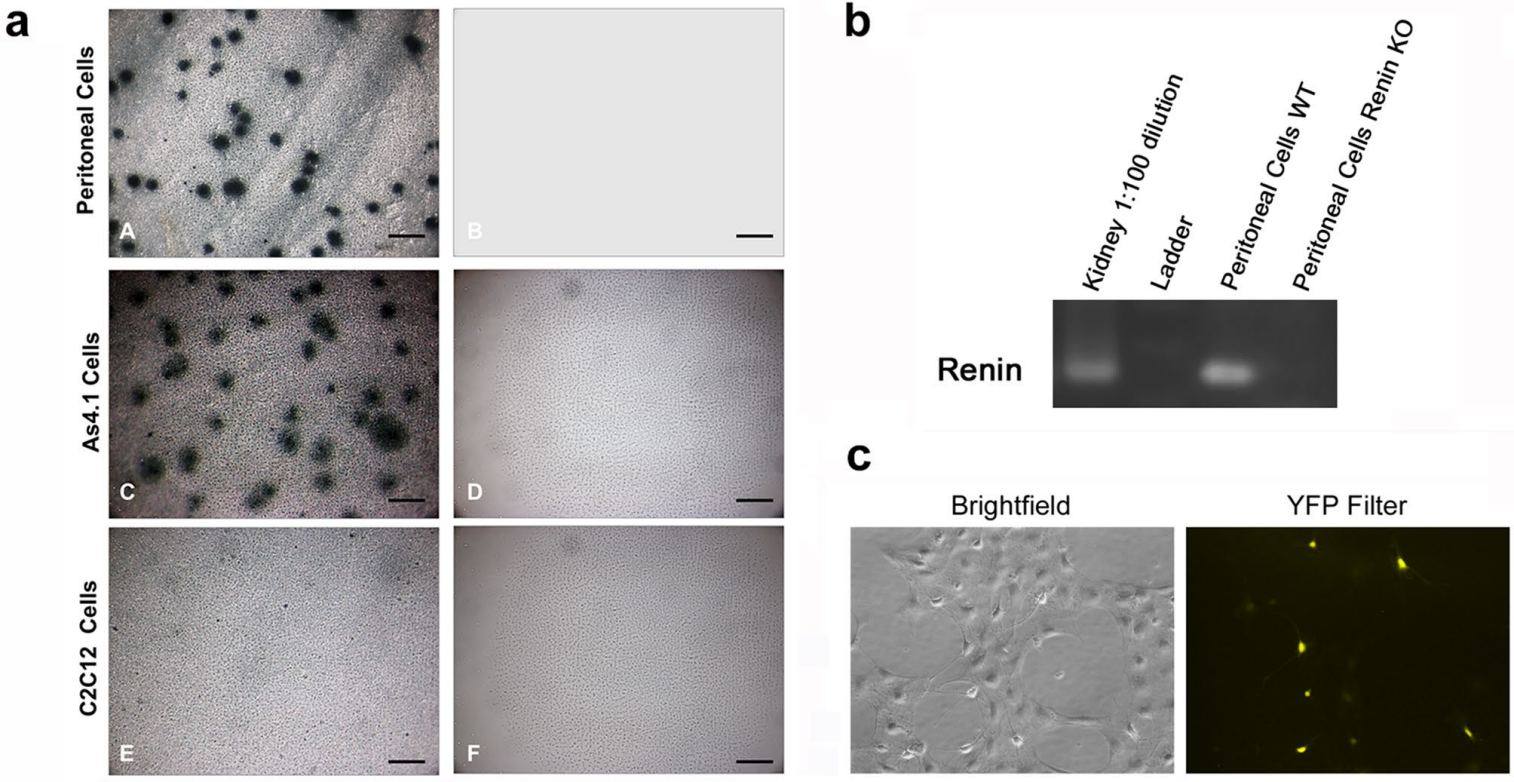
Peritoneal cells express renin. (**a**) A dot membrane immunoassay shows that peritoneal cells derived from the renin lineage appear as dark blue dots, indicating that these cells actively manufacture renin (A). A similar pattern is obtained with As4.1 cells, a mouse tumoral cell line that manufactures renin constitutively (C). By the contrary, no spots were detected in C2C12 cells, skeletal muscle cells that do not normally synthesize renin (E). Further, no spots are detected in any of the cells when the membrane immunoassay is performed in the absence of the primary renin antibody (B, D, F) (scale bars A-F 200 µm). (**b**) Semi-quantitative RT-PCR was performed on wildtype peritoneal cells and peritoneal cells from a renin KO animal. Kidney RNA was used as a positive control. (**c**) Peritoneal cells from Ren1^c-YFP^ reporter mice, where YFP marks active renin expression were grown in culture. YFP was demonstrated by immunofluorescence.

To determine whether these lymphocytes with a renin pedigree manufacture renin protein, we used a dot membrane immunoassay^13,14^. Peritoneal cells from *Ren1*^*dcre/*+^*; mTmG* reporter mice were cultured on nitrocellulose membranes for 48 h followed by dot immunoassay for renin using a well characterized renin antibody and color development detection kit as previously described^15^. Figure 2a shows that peritoneal cells derived from the renin lineage appear as discrete blue dots, indicating that these cells actively manufacture renin. A similar pattern is obtained with As4.1 cells, mouse tumoral cells that produce renin constitutively (Fig. 2a). No spots were detected in C2C12 cells, skeletal muscle cells that do not normally manufacture renin (Fig. 2a). Further, peritoneal cells from WT mice expressed authentic renin mRNA, verified by sequencing, and not a related aspartyl protease (Fig. 2b and Supplementary Fig. S2). Renin mRNA was not present in cells from renin null mice^16^. Consistent with the dot membrane immunoassay results, renin mRNA was present in As4.1 cells but not C2C12 cells (Supplementary Fig. S2). To further corroborate these findings, we obtained peritoneal cells from *Ren1*^*c-YFP*^ mice^17^, where YFP is under the control of the renin super-enhancer^3,18^ and reports the transcriptional activity of the renin gene. Figure 2c shows YFP + cells from the peritoneal cavity, indicating that these cells actively transcribe renin and YFP.

Previous work by other groups has demonstrated unique renin isoforms which are present in various tissues of mice, rats, and humans and differ from the conventional full length renin transcript expressed by JG cells of the kidney^19–21^. These isoforms have alternative transcription initiation sites and lack exon 1 of the full length transcript which encodes for the signaling peptide and a portion of the pro-segment. Thus, these isoforms are not secreted in a regulated manner but instead remain intracellular in an active form. To evaluate the nature of the renin transcript expressed by B-1 cells, we designed primer sets that span the renin gene (Supplementary Fig. S2 and Supplementary Table S2). Using this strategy, we found that the renin isoform expressed by B-1 cells lacks exon 1. Consistent with this result, we found that B-1 lymphocytes do not secrete renin. We obtained B-1 cells from the peritoneal cavity of wildtype mice and cultured these cells for 24 h in DMEM + 5% FBS media. We then performed an enzyme-linked immunoassay (ELISA) for mouse renin (RayBiotech, Peachtree Corners, GA) on the media and cell lysate samples (As4.1 and C2C12 cells served as positive and negative controls respectively). While we could detect intracellular renin protein from B-1 cell lysates (consistent with MIA experiments above), there was no renin protein secreted in the media (Supplementary Table S3).

**Figure 3:**
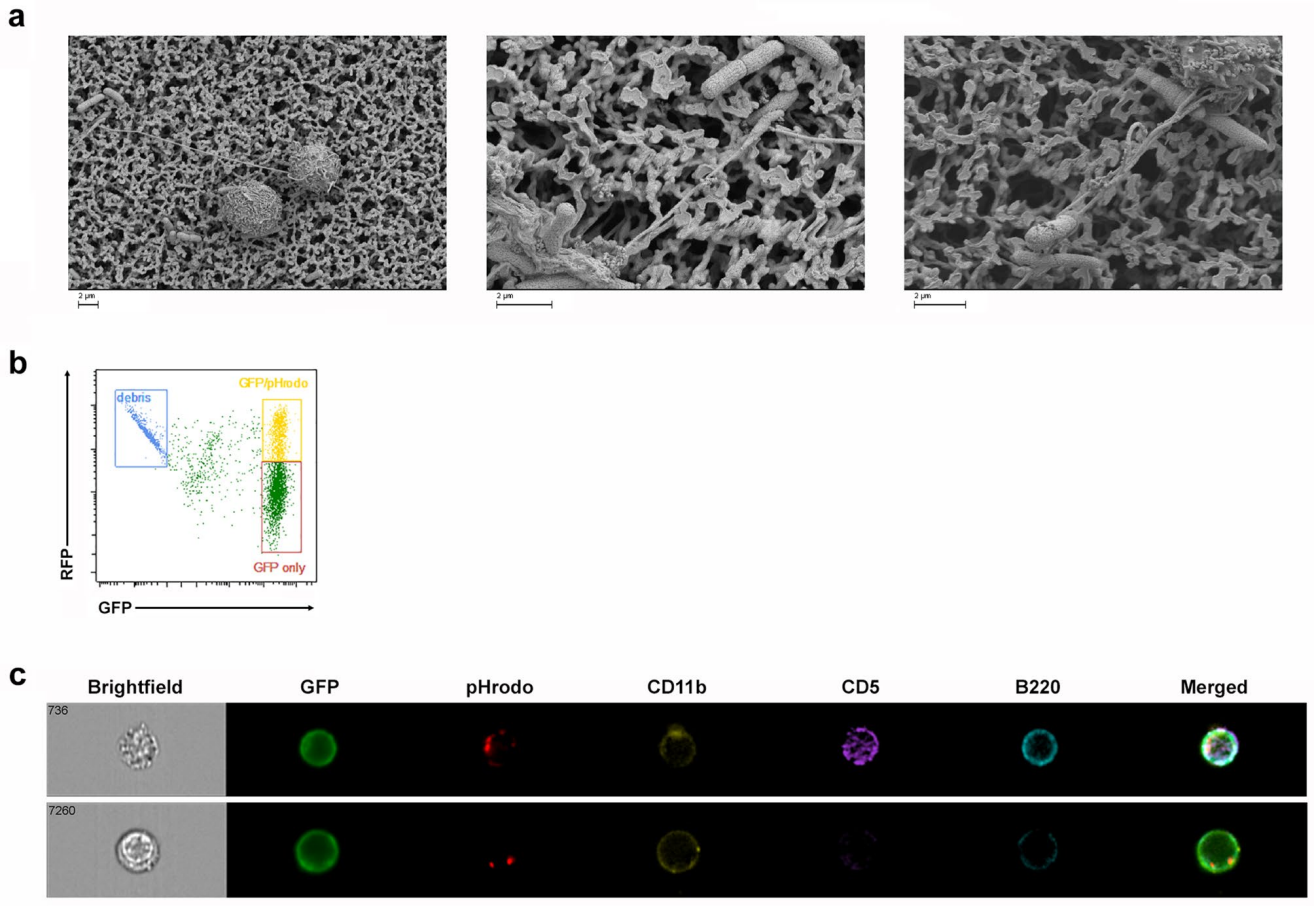
Renin-lineage peritoneal cells physically interact with bacteria and phagocytose bacteria particles. (**a**) Renin-lineage cells were incubated with 042 E. Coli bacteria for 2 h and then processed for scanning electron microscopy. Renin-lineage B-1 cells associate with bacteria through cable-like extensions. (**b**) A representative flow cytometry plot demonstrating GFP^+^ cells (renin lineage cells) which are double positive for pHrodo. (**c**) Identification of renin lineage peritoneal cells that phagocytosed *E. Coli* particles using an ImageStreamX Mark II system. The ImageStream system software (IDEAS) obtained images of events determined to be GFP^+^ cells that have phagocytosed pHrodo. The top panel shows an image of a renin-lineage B-1a B cell which has phagocytosed *E. Coli* particles (pHrodo^+^). This cell expressed CD11b, CD5, and B220. The bottom panel shows an image of a renin-lineage B-1b cell (CD5−).

The primary role of B-1 B cells is to produce natural antibodies which react with foreign antigens as a component of the innate immune system. Therefore, we set out to examine whether renin-bearing GFP^+^ B-1 lymphocytes interact with bacteria. First, we incubated GFP^+^ B-1 lymphocytes with CFP-expressing *E. Coli* and recorded time-lapse pictures. GFP^+^ B-1 lymphocytes trailed the CFP^+^ bacteria and made numerous contacts with them via the assembly and disassembly of pseudopod-like extensions (See Supplementary material, video S1). To explore the nature of the lymphocyte-bacterial contacts, we used scanning electron microscopy. Peritoneal cells emit cable-like extensions and mesh-like structures that entrapped the bacteria which subsequently become immobilized (Fig. 3a). To our knowledge, these poorly understood structures have not been previously described in lymphocytes.

To determine whether renin-lineage B-1 cells phagocyte bacteria, we exposed GFP^+^ B-1 cells to *E. Coli* particles labeled with pHrodo^22^. When the particles are internalized and routed to lysosomes, the low pH in the lysosomes results in red fluorescence emanating from the bacterial particles. Using ImageStream software (IDEAS), we observed those phagocytic events. Figure 3b shows the dot-plot of intensity of GFP (X axis) versus pHrodo (Y axis). There is a double-positive GFP/pHrodo population indicating that a significant proportion of GFP^+^ B-1 cells have ingested the bacterial particles (Fig. 3b, yellow box). Figure 3c shows examples of renin lineage B-1a (top) and B-1b (bottom) lymphocytes which have ingested bacterial particles.

**Figure 4:**
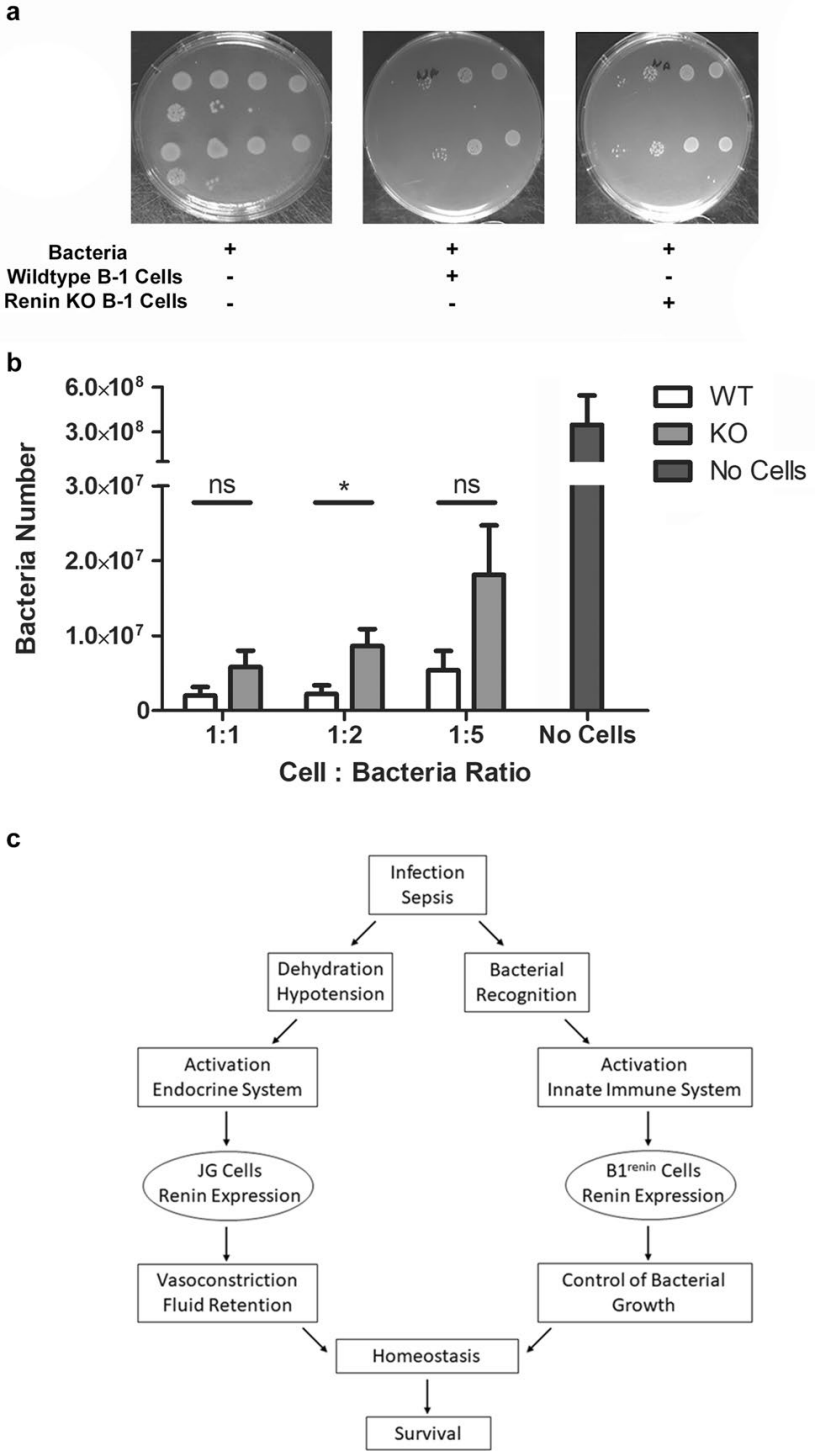
Peritoneal cells from renin KO mice have decreased ability to inhibit bacterial growth in vitro. (**a**) Peritoneal B-1 lymphocytes were obtained from wildtype and renin-KO mice. These cells were co-cultured with *Salmonella typhimurium* bacteria for 2 h. A third group included *Salmonella typhimurium* bacteria alone. Cells were removed after 2 h, and the bacteria were grown in agar for 24 h. Bacteria numbers were then calculated. (**b**) These experiments were repeated at cell:bacteria ratios of 1:1, 1:2, and 1:5. Each experiment was performed 5 times, and the results were grouped together. Statistical significance was determined using the Mann–Whitney test as values represented paired observations (WT vs KO 1:2 cell:bacteria ratio, P = 0.0362). (**c**) Schematic as to how the endocrine renin-angiotensin system and the innate immune system coordinate efforts in response to threats to homeostasis.

To determine whether renin-expressing B-1 lymphocytes possess bactericidal activities, we incubated them with *Salmonella typhimurium* (ATCC14028) for two hours and measured bacterial growth over 24 h by bacterial counting of colony forming units. Incubation of bacteria with B-1 cells markedly diminished the number of colonies compared to bacteria growing in the absence of B-1 cells, indicating that B-1 cells possess the ability to inhibit bacterial growth (Fig. 4a). To explore whether renin produced by B-1 cells contributed to the antimicrobial function, we cultured peritoneal B-1 lymphocytes from renin null (*Ren1*^*c*−*/*−^) mice and from wild-type controls with *Salmonella* at various cell : bacteria ratios for two hours. Bacteria co-cultured with wildtype B-1 cells had decreased growth. However, when bacteria were co-cultured with renin null B-1 cells, the inhibition of bacterial growth was markedly diminished (Fig. 4a,b) suggesting that intracellular renin may play a role in defending against bacterial pathogens. To investigate if renin facilitates bacterial killing as part of a local renin-angiotensin system (RAS) by enzymatically cleaving angiotensinogen to angiotensin, we looked for other components of the RAS within B-1 cells. We found no expression of angiotensin-converting enzyme (ACE), angiotensinogen (Atg), or the ANG II type 1 receptor (AT_1_) within B-1 cells from the peritoneal cavity, although it is possible they can be taken up from the circulation (Supplementary Fig. S3). To further investigate if the enzymatic activity of renin is responsible for inhibiting bacteria growth, we repeated our bacteria co-incubation assay in the presence of exogenous renin (via either As4.1 cells or by adding recombinant renin, (Sigma-Aldrich, St. Louis, MO)) or the renin inhibitor Aliskiren^23–26^ (Sigma-Aldrich, St. Louis, MO). Neither exogenous renin nor renin inhibition affected bacterial clearing (Supplementary Fig. S4), suggesting that the ability to inhibit bacterial growth is not due to the extracellular enzymatic activity of secreted renin but likely due to the action of the intracellular enzyme. This hypothesis remains to be examined in detail.

Altogether, these studies uncovered an unsuspected, previously unrecognized role of renin-expressing cells in the defense of the organism against infections.

## Discussion

We have previously shown that progenitor cells from different embryonic layers express renin during development^1^. Those progenitors differentiate into a variety of phenotypically and functionally diverse group of cells distributed throughout the body. Whereas most of them stop producing renin, the kidney juxtaglomerular cells and the B-1 lymphocytes retain the ability to synthesize renin in adult life^1^. Interestingly, these two cell types share some core transcriptional regulators^4^. We have previously shown that deletion of RBP-J in the mouse kidney alters the fate of renin cells. As a result, the animals are unable to maintain blood pressure when exposed to a threat to homeostasis^27^. Similarly, deletion of RBP-J in pre-B lymphocytes leads to inability of the cells to differentiate resulting in their uncontrolled neoplastic proliferation^4^. Kidney juxtaglomerular cells and B-1 lymphocytes may also share functional responses. For instance, administration of captopril which decreases blood pressure in adult mice, results in an increase in the number of renin producing cells both in the kidney and in the bone marrow^4^ suggesting that these two seemingly distant cells act in concert to maintain homeostasis when confronted with a threat to survival. A further look at these cells indicate them to be engaged in the unique specialized control of homeostatic defense. JG cells in the kidney located at the vascular pole of glomeruli are strategically located to sense and respond to changes in the composition and volume of the extracellular fluid and to changes in blood pressure. Similarly, B-1 lymphocytes recognize and react to the presence of foreign antigens and microorganisms. Figure 4c illustrates how these cells may coordinate their response upon a threat to survival, as it frequently occurs in gastrointestinal or abdominal infections. Under these circumstances, hypotension and volume depletion induces JG cells to release renin to the circulation leading to the generation of angiotensin(s), vasoconstriction, sodium chloride reabsorption and reestablishment of fluid electrolyte and blood pressure balance^28^. Similarly, peritoneal B-1 renin cells which have the capability to recognize, entrap, phagocyte and kill bacteria, act as an early line of defense to counteract and stop the threat. Multiple studies have shown that peritoneal B-1 cells possess anti-bacterial properties^29–31^. Interestingly, the presence of renin renders B-1 lymphocytes more effective in bacterial killing, suggesting that intracellular renin may facilitate phagocytosis, degradation of bacterial components, and bacterial inhibition.

Several recent studies have implicated the renin-angiotensin system in the regulation and function of the immune system in addition to its classical role in fluid, electrolyte, and blood pressure control. Individual components of the RAS, including enzymes and receptors, have been shown to have independent functions within the native and adaptive immune system^32^. For example, the (pro)renin receptor (PRR) is required for normal T lymphocyte development^33^. And the enzyme ACE has been shown to enhance the antibacterial effectiveness of neutrophils by increasing the production of reactive oxygen species^34^. ACE does not directly kill bacteria through its enzymatic activity but instead modifies the phenotype of neutrophils to enhance their immune effectiveness. When combined, the independent effects of these individual components expand the reach of the whole RAS to another defensive function beyond fluid-electrolyte control.

In this work, we found that the renin promoter is activated in B-1 B cells, a unique renin transcript is expressed within B-1 cells, renin protein is present within B-1 B cells but not secreted, and the presence of intracellular renin facilitates bacterial killing. Although the exact mechanism is not known, several possibilities exist. First, renin expression may contribute to intracellular angiotensin generation as occurs in the brain^20,35–38^. Second, intracellular renin may modulate mitochondrial metabolism and protect against accumulation of reactive oxygen species as occurs in cardiomyoblasts^39,40^. Third, renin may function in an autocrine manner on B-1 cells, enhancing their phagocytosis ability, similar to how ACE expression enhances the immune function of neutrophils^34,41,42^. Finally, intracellular renin may work by degrading bacterial components within lysosomes. Further work will be required to answer these questions. Pharmacologic targeting of renin with Aliskiren is a starting point, however this drug only inhibits the enzymatic activity of renin. Other strategies, including siRNA, will be important to target additional (unknown) functions of cytosolic renin.

In summary, renin, and the diverse group of cells that synthesize it, are at the epicenter of two systems crucial for survival: the endocrine renin-angiotensin system driven to maintain cardio-circulatory homeostasis by regulating volume and tissue perfusion and the innate immune system designed for the rapid control of infections.

## Methods

### Mice

*Ren1*^*dCre/*+^ mice express Cre recombinase in renin cells^6^. *Ren1*^*dcre/*+^ mice were bred to the lineage reporter line *mT/mG*, in which non-recombined cells express mTd, and Cre-mediated recombined cells express GFP^43^. Thus, GFP is expressed in Ren1-expressing cells and all descendants. Certain inbred strains of mice carry a duplicated copy of the renin structural gene, Ren2^44^. Ren1^d^ and Ren2 have ~ 97% homology and are both expressed within the kidney in two-gene strains of mice^44,45^. In our work, *cre recombinase* replaces the Ren1^d^ gene and lineage tracing studies faithfully report the expression and lineage of Ren1^d^ but not Ren2^6^. *Ren1*^*c-YFP*^ transgenic mice express YFP in cells that actively express renin and were used to identify cells that actively express renin^17^. *Ren1*^*c*−*/*−^ mice are renin knockout mice, and wildtype (“WT”) mice are C57BL/6. All mice used in experimental procedures were > 3 months old.

### Cells

Peritoneal cells were obtained from wildtype, renin knockout (*Ren1*^*c*−*/*−^), renin-expressing (*Ren1*^*c-YFP*^), and renin-lineage (*Ren1*^*dCre/*+^*;mTmG*) mice. Briefly, mice were anesthetized, and scissors and forceps were used to remove the skin overlying the ventral portion of the peritoneum. 5 mL of PBS with 3% FBS was injected into the peritoneal cavity with a 30 gauge needle. The peritoneum was then massaged for 5 min to mobilize cells into the PBS solution. An 18 gauge needle was then used to remove the fluid containing peritoneal cells. The cell suspension was then pelleted and resuspended in DMEM + 5% FBS media. B-1 cells were isolated using the protocol described by Popi et al.^46^. Briefly, peritoneal cells were plated on 6 well plates and incubated at 37 °C in 5% CO2 for 60 min. After 60 min, the supernatant was discarded, and the adherent cells were gently removed using a cell scraper. As4.1 cells are a renin-expressing tumoral cell line (ATCC, CRL-2193)^47^. C2C12 cells are an immortalized mouse myoblast cell line (ATCC, CRL-1772).

### Bacteria

DH5α E. coli bacteria were co-cultured with peritoneal cells from *Ren1*^*dCre/*+^*;mTmG* mice. DH5α bacteria were transformed with a CFP plasmid (Oxford Genetics Ltd, Oxfordshire, United Kingdom) using Kanamycin selection (1 µl of CFP plasmid added to 50 µl bacteria). Colonies positive for CFP were selected. These bacteria were subsequently termed “DH5α-CFP”. DH5α-CFP bacteria were co-incubated with peritoneal cells, and time-lapsed pictures were taken. 042 bacteria are a strain of enteroaggregative *Escherichia coli*^48^. These bacteria were used for scanning electron microscopy (SEM) (below). For bacterial ingestion and killing assays, we used *Salmonella typhimurium* (ATCC 14,028) and modified the protocol as described^49,50^. Briefly, we isolated peritoneal cells from WT or renin KO mice. Peritoneal cells were plated in a 6 well dish in DMEM + 5% FBS for 60 minutes^46^. The floating cells were removed, and the adherent cells were isolated using a cell scraper. Cells were then incubated with Salmonella at various cell:bacteria ratios (as indicated in the text) for 2 h at 37 °C. After 2 h, cells were lysed with 0.02% SDS/PBS, and bacteria were transferred to Eppendorf tubes. Bacteria were then plated in agar dishes and grown overnight, and bacteria colony forming units were quantitated.

### Statistics

Statistical significance between groups was evaluated using the Mann–Whitney test. Differences were considered statistically significant at *P < 0.05 levels. Bar graphs are expressed as mean ± s.e.m.

### Flow cytometry

To characterize the immunophenotype of renin-lineage cells from reporter mice, flow cytometry analysis was performed. Single cell suspensions were obtained from the bone marrow, spleen, peripheral blood, and peritoneal fluid as previously described^51^. Femurs were isolated and flushed with PBS + 5% fetal bovine serum (FBS), and cells were passed through a 70 µm cell strainer. Spleens were passed through a 70 µm cell strainer using a syringe plunger to gently disrupt the tissues. Cells were rinsed through the strainer with PBS + FBS. Cell suspensions were treated with red blood cell lysis buffer, resuspended in PBS + FBS, counted using the Cellometer mini (Nexcelcom Bioscience, Lawrence, MA, USA) and distributed into microcentrifuge tubes for antibody labeling against surface markers (Supplementary Table S1)^51^. Antibodies were added at predetermined concentrations and incubated at room temperature for 20 min. Immunophenotyping was performed in the University of Virginia Flow Cytometry Core laboratory using a Fortessa cytometer, and data were analyzed with the FCSExpress program (De Novo Software, Los Angeles, CA, USA). For embryos, single cell suspensions were obtained from the yolk sac, fetal liver, and fetal spleen by passing tissues through a cell strainer as described above.

### RNA extraction and polymerase chain reaction analysis

Total RNA was isolated from the yolk sacs and livers of mouse embryos using Trizol extraction (Life Technologies, Grand Island, NY, USA) according to manufacturer’s instructions, as previously described^51^. Complementary DNA (cDNA) was prepared from 2 µg RNA using Maloney murine leukemia virus reverse transcriptase (Life Technologies) and an oligo(dT) primer according to the manufacturer's instructions. PCR was performed on 2 µl cDNA using Taq DNA polymerase (Promega, Madison, WI, USA) in an Eppendorf thermocycler^51^.

### Transplant studies

Fetal liver cells from *Ren1*^*dcre/*+^*;mTmG* mice were isolated (as above) and transplanted into adult WT mice after being treated with radiation (13 Gy divided into 2 fractions) via tail vein injection. Adult recipient mice were sacrificed 2 weeks later, tissues were harvested, and the percentage / identity of renin-lineage cells were determined by flow cytometry.

### Membrane immuno assay (MIA)

This was performed by culturing cells on a nitrocellulose membrane^13^ followed by an immunoassay for renin modified based on the dot immunoassay protocols described previously^14^. Briefly, cells were grown in low density on 0.22µ nitrocellulose membranes (Bio Rad) and cultured for 48 h at 37 °C/5% CO_2_ in six well plates. Cells were fixed on the membranes by baking them at 100 °C for 30 min followed by 2% PFA treatment for 30 min at room temperature (RT). The membranes were subsequently immune assayed for renin with all the steps performed with gentle shaking at RT. After fixing, membranes were washed with 0.1 M Tris buffer; pH 7.4 + 0.05% tween (Buffer I) and further treated with 3% hydrogen peroxide for 15 min to quench the endogenous peroxide activity. Blocking was performed with 3% protease free bovine serum albumin (BSA) in 0.1 M Tris buffer (pH7.4) for an hour. Primary antibody for renin (affinity purified rabbit polyclonal custom made in our laboratory^52^) at 1:2500 dilution in buffer I was added to the membrane and incubated overnight at RT. A biotinylated anti-rabbit secondary antibody kit **(**Vectasatin–Elite, ABC Reagent kit, Vector Laboratories) was used to detect and amplify the primary antibody signals, following the manufacturer’s protocol. After treating with avidin–biotin solution, membranes were washed in buffer I and placed in a peroxidase-free chamber containing tetramethylbenzadine warmed to RT (TMB membrane peroxidase substrate solution, Kirkeguard and Perry Laboratories) and stored in dark.

### Immunofluorescence

Immunofluorescence was performed on frozen sections. Imaging of GFP^+^ cells from various tissues was done using a Leica DFC310 FX digital camera connected to a Leica DFC 480 microscope.

### SEM

Peritoneal cells were isolated from reporter mice and cultured in a 6 well plate with 20 × 20 mm coverslips in the wells. Peritoneal cells were incubated for 1 h, and then the supernatant was removed. 500 µl of 042 bacteria were added to each well, and the cells / bacteria were incubated for 2 h. Plates were then centrifuged at 1400 RPM for 10 min, and the supernatant was removed. 4% paraformaldehyde was added to fix the cells / bacteria, and samples were stored at 4ºC overnight. Scanning electron microscopy was performed by the University of Virginia Microscopy Core Facility.

### Phagocytosis assay

Peritoneal cells were collected from *Ren1*^*dcre/*+^*;mTmG* mice as described above. Cells were plated in a 6 well plate and incubated for 90 min. pHrodo E. coli BioParticles (Life Technologies, Eugene, OR, USA) were added to the wells and incubated for 2 h. Cells were then collected from the wells, washed with PBS + FBS, centrifuged, and resuspended. Aliquots were labelled with antibodies (above) for flow cytometry analysis.

### Renin protein concentration

Renin protein concentration from cell lysates and from cell media was determined using ELISA following the manufacturer’s instructions (RayBiotech).

### Study approval

All studies were performed in accordance with the National Institutes of Health Guide for the Care and Use of Laboratory Animals and were approved by the Institutional Animal Care and Use Committee of the University of Virginia (Protocol No. 2433, Initial Review Data 6/12/2018, First Annual Review Date 6/11/2019). All animal experiments were conducted in compliance with the Animal Research Reporting In Vivo Experiments (ARRIVE) guidelines^53^.

## Supplementary Information

Supplementary Information 1.

Supplementary Information 2.

## Data Availability

All data generated or analysed during this study are included in this published article (and its Supplementary Information files).
