# Supplementary material for: A primitive type of renin-expressing lymphocyte protects the organism against infections
Source: Sci Rep. 2021 Mar 31;11:7251. doi: 10.1038/s41598-021-86629-w (PMC8012387; doi:10.1038/s41598-021-86629-w)
Supplement: Supplementary file 2 — Supplementary Information 2. [file 41598_2021_86629_MOESM2_ESM.docx]

**A primitive type of renin-expressing lymphocyte protects the organism against infections**

Brian C. Belyea, Araceli E. Santiago, Wilson A. Vasconez, Vidya K. Nagalakshmi, Fang Xu, Theodore C. Mehalic, Maria Luisa S. Sequeira-Lopez and R. Ariel Gomez.

Child Health Research Center, Department of Pediatrics, University of Virginia School of Medicine, Charlottesville, VA, U.S.A.

**Correspondence:**

R. Ariel Gomez ([rg@virginia.edu](mailto:rg@virginia.edu)) and Maria Luisa S. Sequeira-Lopez ([msl7u@virginia.edu](mailto:msl7u@virginia.edu))

**Supplementary Figure 1. Transplant studies confirm the fate of renin-expressing progenitors during fetal life.**


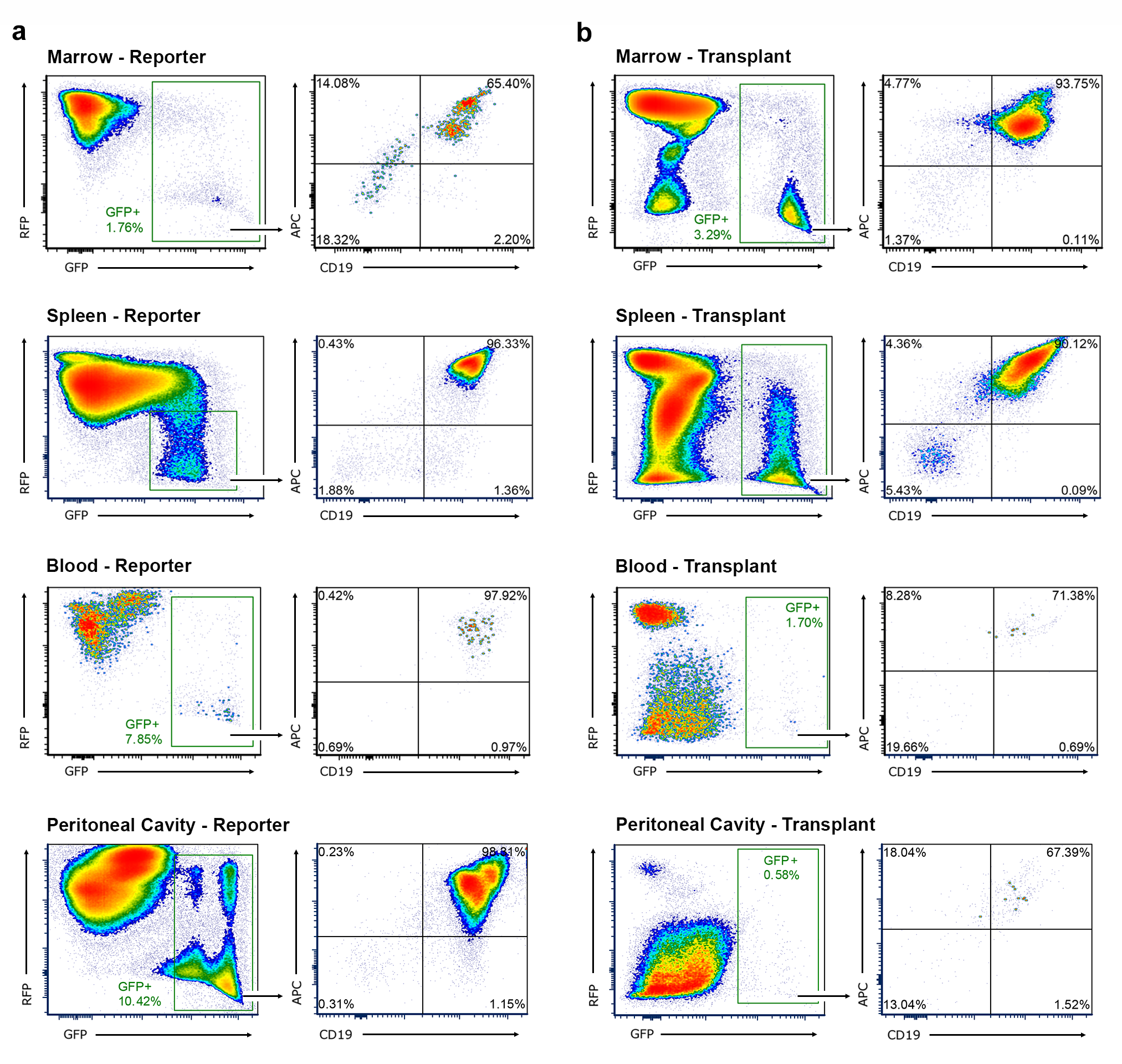


a. Renin-lineage (GFP^+^) cells were isolated from the bone marrow, spleen, blood, and peritoneal cavity of adult reporter mice (*Ren1^dcre/+^; mTmG*). These cells were determined to be positive for the B cell surface markers CD19 and B220. In these figures, APC is the fluorochrome that marks B220. GFP marks cells that have expressed renin, and RFP marks cells that have not expressed renin.

b. Fetal livers were isolated from E16.5 *Ren1^dcre/+^;mTmG* embryos (where GFP marks renin-expressing cells and descendants). These cells were injected into the tail vein of irradiated adult wildtype hosts. Transplant recipients were studied 3 weeks after transplant, and engraftment was determined by the percent of GFP/RFP in the host organs. There was excellent engraftment in the bone marrow, but reduced engraftment in peripheral tissues. In all tissues, the transplanted renin lineage (GFP^+^) cells mirrored renin lineage cells in adult reporter mice, expressing CD19 and B220.

**Supplementary Figure 2. PCR Evaluation of Renin Isoform in B-1 B Cells**


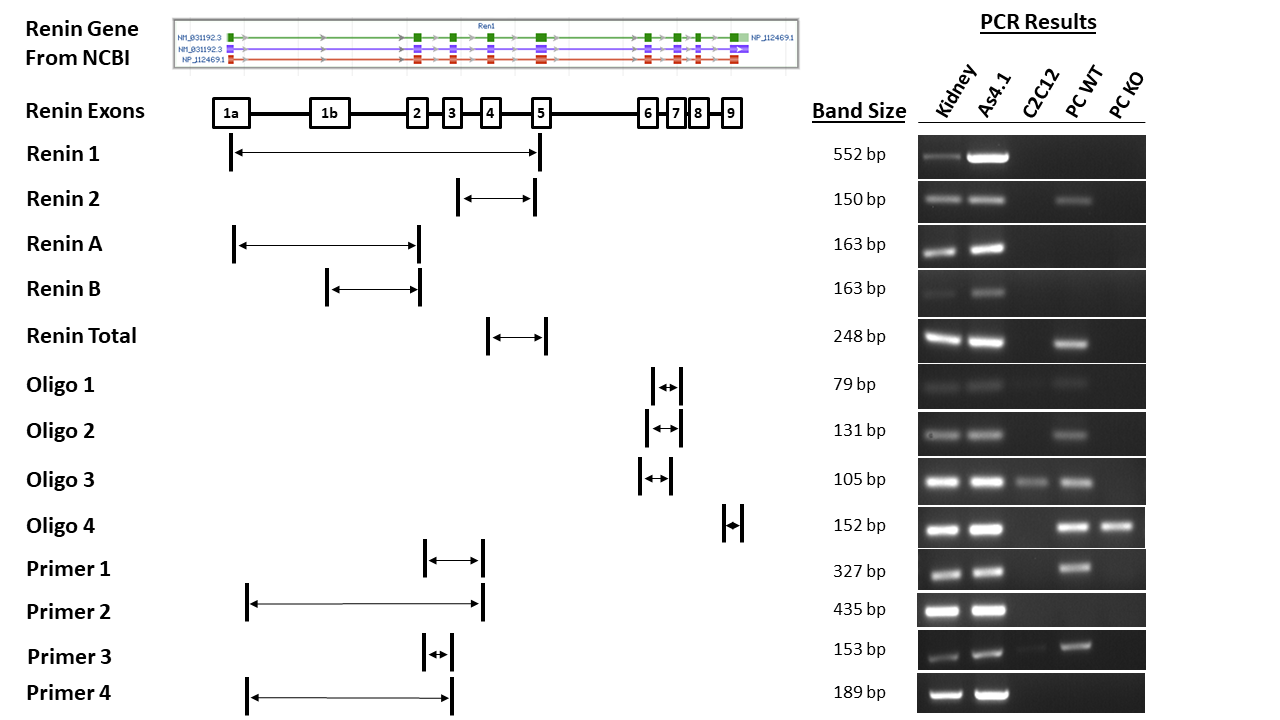


We performed a series of PCR reactions to evaluate the nature of the renin isoform expressed by B-1 lymphocytes. The mouse renin gene (from NCBI) is displayed at the top followed by a graphic showing the location of renin exons (including the alternative exon 1b which is within the first intron). On the left are the designated primer names, followed by the locations of the forward and reverse primers (marked by vertical black bars) for each primer pair. The semi-quantitative PCR results are displayed on the right side of the figure. cDNA from newborn kidney tissue and As4.1 cells were used a positive control. cDNA from C2C12 skeletal muscle cells and peritoneal cells from renin knockout mouse were used as negative controls. We found that within B-1 lymphocytes, exons 2-9 are expressed. However, exon 1 and exon 1b are not expressed in B-1 cells. There is an expected band in B-1 cells from renin knockout mice for exons 8 and 9, as this portion of the renin gene was not deleted.


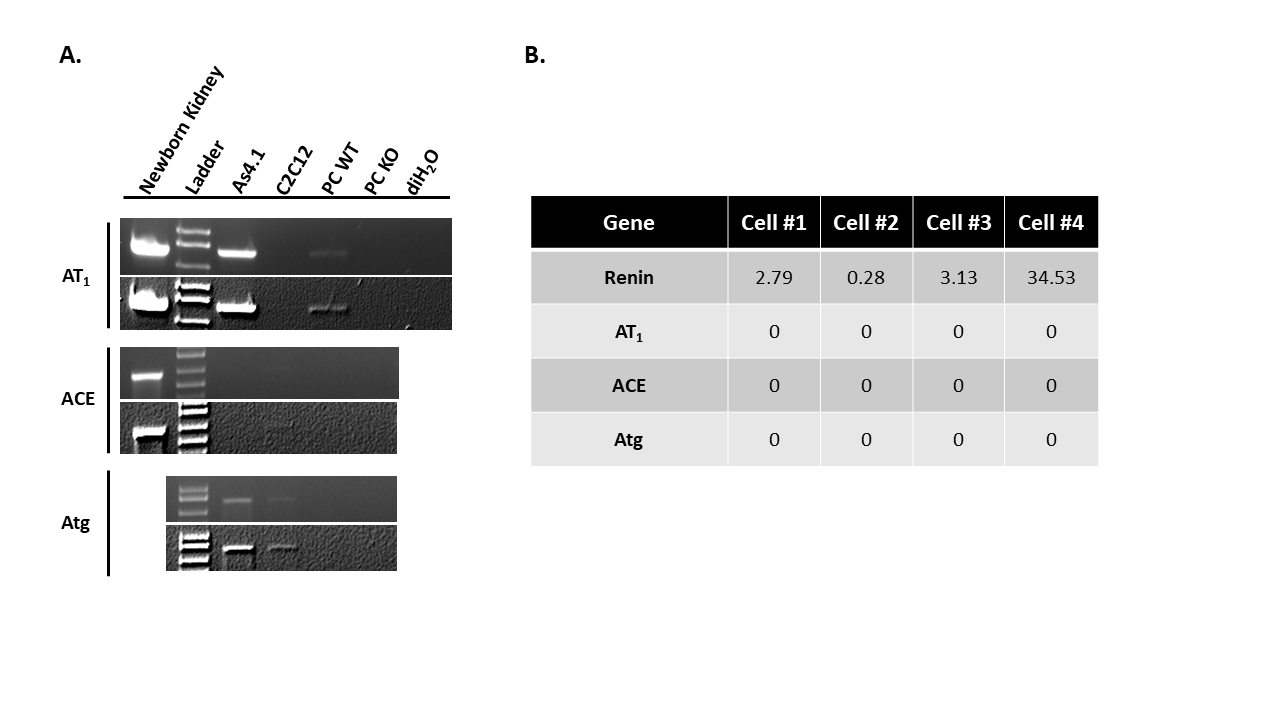


**Supplementary Figure 3. Evaluation of RAS Components in B-1 B Cells**

We performed semi-quantitative PCR on B-1 cells from wildtype mice to evaluate for the presence of components of the RAS. We found that B-1 peritoneal cells (“PC WT”) have expression of AT_1_ but not ACE or Atg.


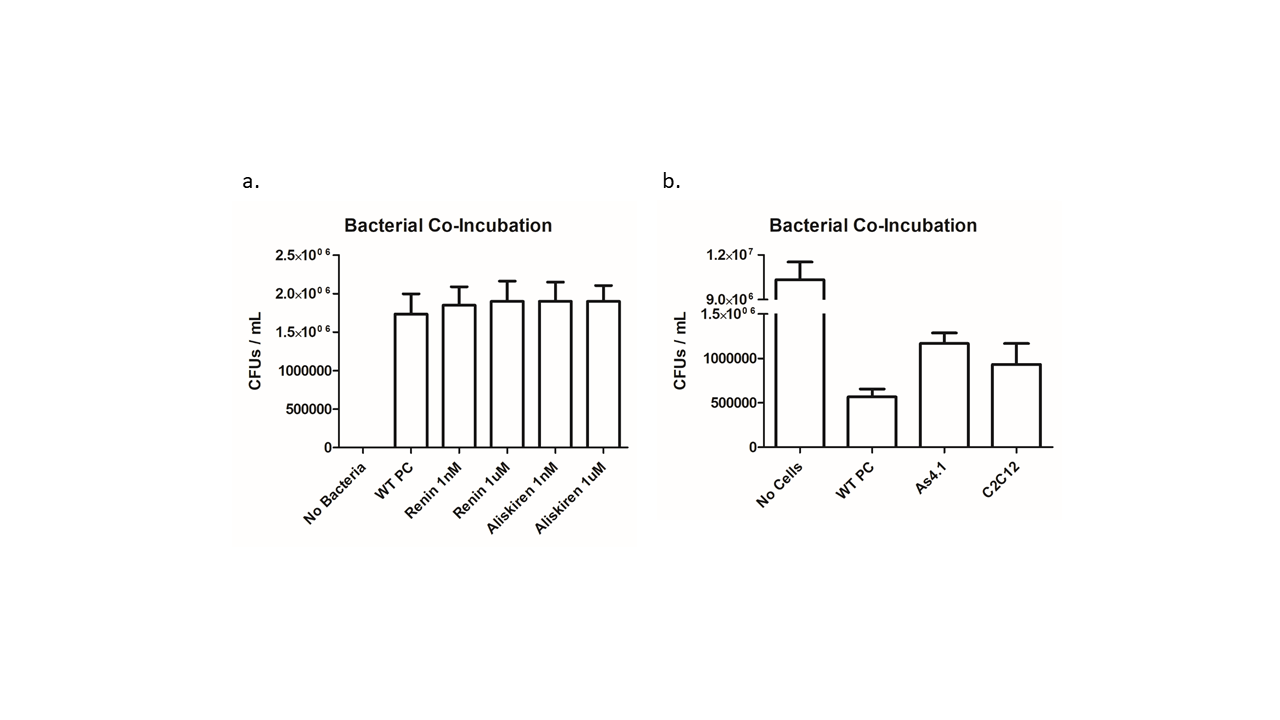
**Supplementary Figure 4. Bacteria Co-Incubation Studies with B-1 Lymphocytes**

a. B-1 B lymphocytes were co-incubated with Salmonella bacteria in the absence or presence of exogenous Renin or the renin inhibitor Aliskiren. There was no difference in bacteria growth between any of these groups. This experiment was performed two times with 3 experimental replicates in each experiment.

b. Salmonella bacteria were co-incubated with B-1 cells from the peritoneal cavity of wildtype mice (“WT PC”), As4.1 cells and C2C12 cells. This experiment was performed a single time with 3 experimental replicates.

**Supplementary Table 1. Antibodies Used for Flow Cytometry**

| **Antibody** |  | **Fluorochrome** | **Concentration** |
| --- | --- | --- | --- |
| CD19 | Pan B cell marker expressed on both B-1 and B-2 B cells | APC | 0.75 µg per 10^6^ cells |
| B220 | Pan B cell marker expressed highly on B-2 B cells and more dim on B-1 B cells | APC / Cy7 | 1 µg per 10^6^ cells |
| CD11b | Expressed on B-1 B cells | PerCP / Cy5.5 | 1 µg per 10^6^ cells |
| CD5 | Expressed on B-1a B cells but not B-1b B cells | Brilliant Violet 421 | 5 µl per 10^6^ cells |
| CD43 | Expressed on Pro-B cells and B-1 B progenitor cells | PE / Cy7 | 0.25 µg per 10^6^ cells |
| CD23 | Expressed on mature B-2 Bcells but not on B-1 B cells | PE / Cy7 | 0.25 µg per 10^6^ cells |
| Lineage Cocktail | Cocktail of antibodies against mature hematopoietic cells including B cells, T cells, Granulocytes, Monocytes, NK cells and Erythrocytes | Brilliant Violet 421 | 20 µl per 10^6^ cells |

Abbreviations:

APC = allophycocyanin

Cy7 = cyanine 7

Cy5.5 = cyanine 5.5

PE = phycoerythrin

**Supplementary Table 2. PCR Primers for Supplementary Figure 2.**

| **Primer Name** | **Primer Sequence** |
| --- | --- |
| Renin 1 Forward | 5’-ATGCCTCTCTGGGCACTCTT-3’ |
| Renin 1 Reverse | 5’-GTCAAACTTGGCCAGCATGA-3’ |
| Renin 2 Forward | 5’-TGCTTGTGGGATTCACAGCCTCTA-3’ |
| Renin 2 Reverse | 5’-TGTGCTACAGTGATTCCACCCACA-3’ |
| Renin A Forward | 5’-ACCTTCAGTCTCCCAACACGCACC-3’ |
| Renin A Reverse | 5’-GGGAGGTAAGATTGGTCAAGGAAGG-3’ |
| Renin B Forward | 5’-TTTGATGAGAGGATACGCATAGCACTTC-3’ |
| Renin B Reverse | 5’-GGGAGGTAAGATTGGTCAAGGAAGG-3’ |
| Renin Total Forward | 5’-GTCCGACTTCACCATCCACTAC-3’ |
| Renin Total Reverse | 5’-AGAACACTTCCTCCTTTAGCAC-3’ |
| Oligo 1 Forward | 5’-CTGACTCCTGGCAGATCACG-3’ |
| Oligo 1 Reverse | 5’-ACTACCGCACAGCCTTCTTC-3’ |
| Oligo 2 Forward | 5’-TAGCGACCCGCAGCATTATC-3’ |
| Oligo 2 Reverse | 5’-CTACCGCACAGCCTTCTTCA-3’ |
| Oligo 3 Forward | 5’-AGGTGGTGCTAGGAGGTAGC-3’ |
| Oligo 3 Reverse | 5’-CAGACACCCCCTTCATCGTG-3’ |
| Oligo 4 Forward | 5’-ACAATCGCATTGGATTCGCC-3’ |
| Oligo 4 Reverse | 5’-GGGCAAACACTCGTTAGGGT-3’ |
| Primer 1 Forward | 5’-TGCCTTCTGTCCGGGAATC-3’ |
| Primer 1 Reverse | 5’-CACGGGGGAGGTAAGATTGG-3’ |
| Primer 2 Forward | 5’-GCACCGCTACCTTTGAACGA-3’ |
| Primer 2 Reverse | 5’-ATCTCGCCGTAGTACTGGGT-3’ |
| Primer 3 Forward | 5’-CCTTTGAACGAATCCCGCTC-3’ |
| Primer 3 Reverse | 5’-GTGAAGTCGGACCCGTTCTC-3’ |
| Primer 4 Forward | 5’-GCACCGCTACCTTTGAACGA-3’ |
| Primer 4 Reverse | 5’-TGGATGGTGAAGTCGGACCC-3’ |

**Supplementary Table 3. Renin Concentration in As4.1, C2C12, and B-1 Cell Lysates and Media**

|  | Cell Lysates | Media |
| --- | --- | --- |
| As4.1 cells | 127, 381 +/- 20,752 pg/mL | 2,618.6 +/- 187.6 pg/mL/hour |
| C2C12 cells | 0 | 0 |
| B-1 B cells | 212.4 +/- pg/mL | 0 |

**Full edited gel for Figure 1b**


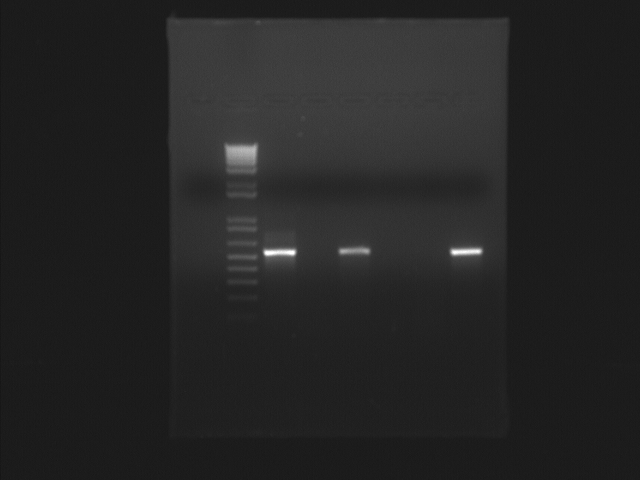


1. 1 kb ladder
2. Newborn kidney
3. E11.5 fetal liver
4. E11.5 yolk sac

1. **2. 3. 4.**

**Lanes shown in Figure 1b**

1. 1 kb ladder
2. Newborn kidney
3. E11.5 fetal liver
4. E11.5 yolk sac

**Full unedited gel for Figure 2b**


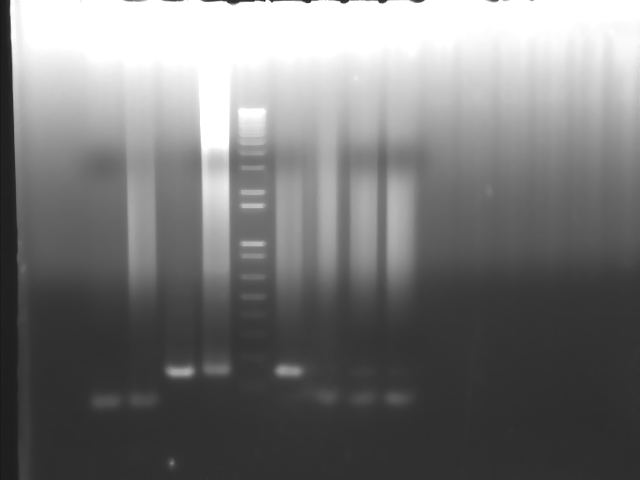


1. **2. 3. 4.**

**Lanes shown in Figure 2b**

1. Newborn Kidney, 1:100 dilution
2. 1 kb ladder
3. Peritoneal cells from wildtype mouse
4. Peritoneal cells from renin KO mouse

**Full unedited gels for Supplementary Figure 2**

Renin 1


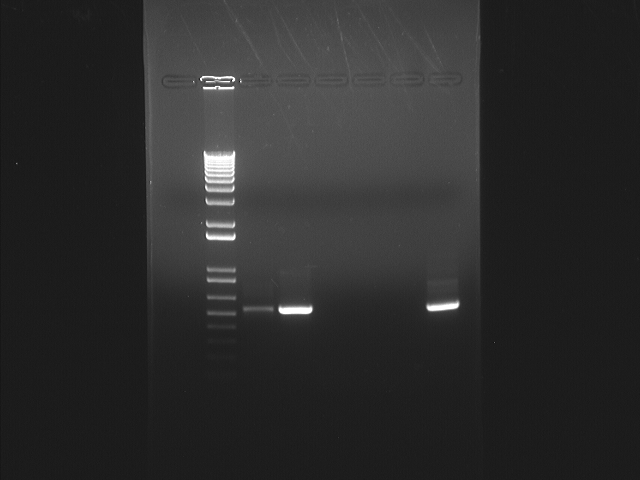


Renin 2


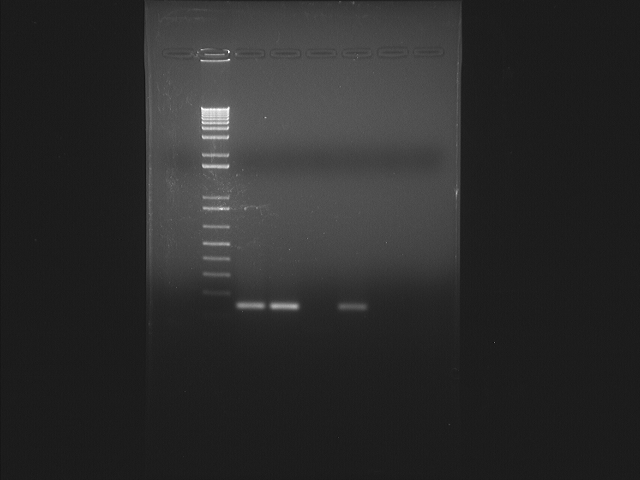


Renin A (left side) and Renin B (right side)


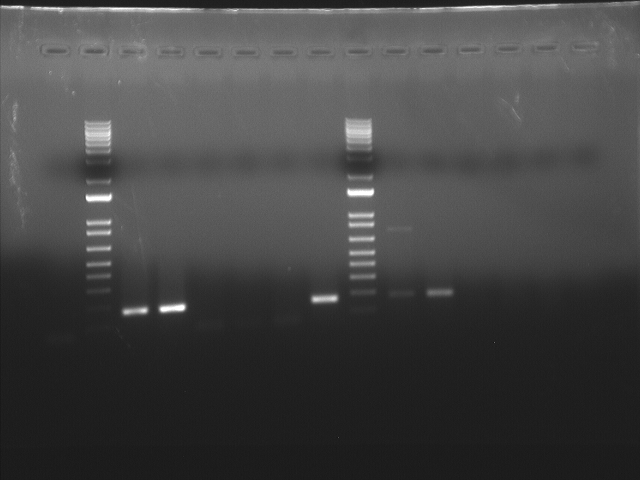


Renin Total


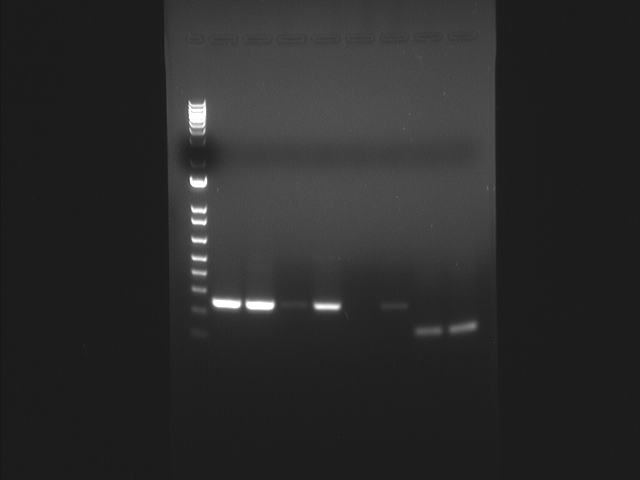


Oligo 1


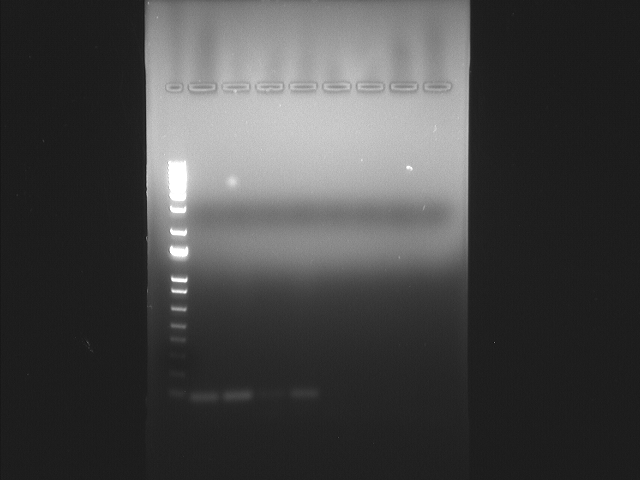


Oligo 2


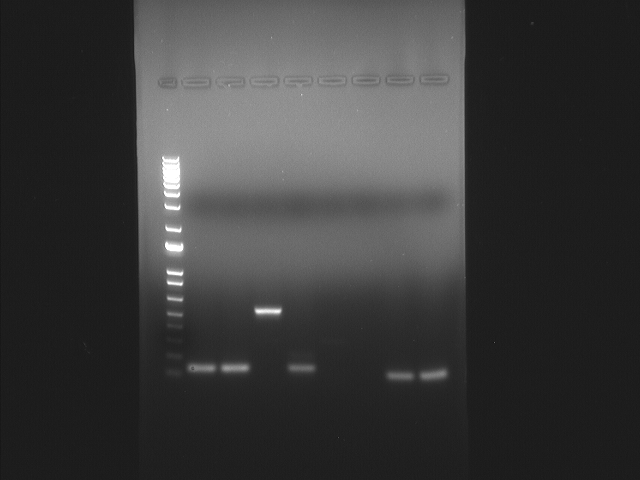


Oligo 3 (middle) and Oligo 4 (left)


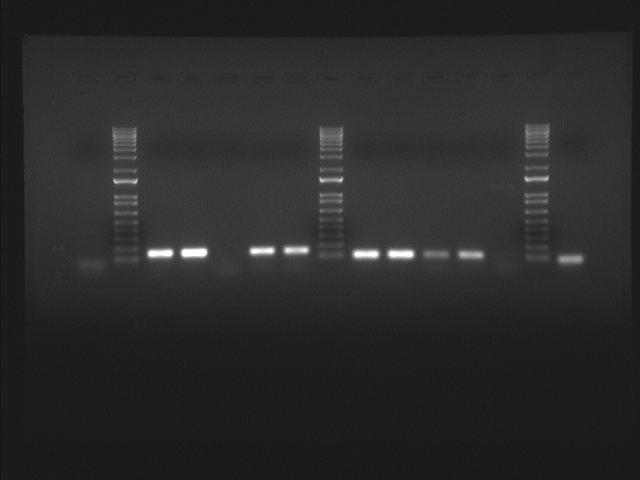


Primer 1 (left side of gel) and Primer 2 (middle)


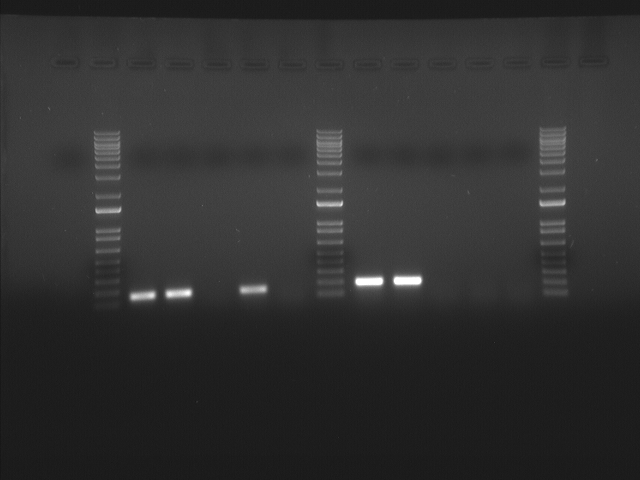


Primer 3 (left side of gel) and Primer 4 (middle)


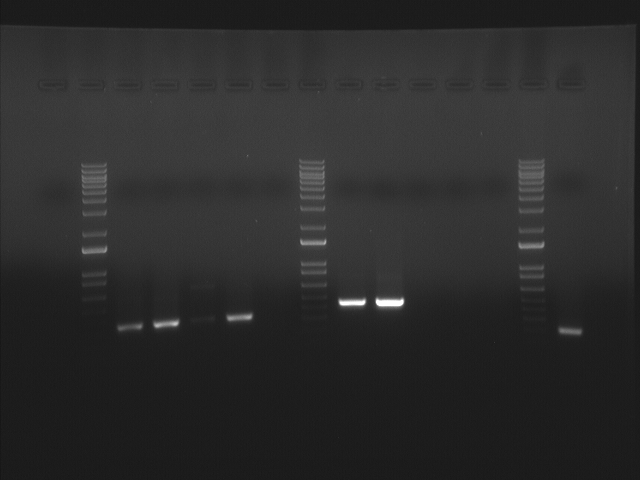


**Full unedited gels for Supplementary Figure 3**

AT_1_


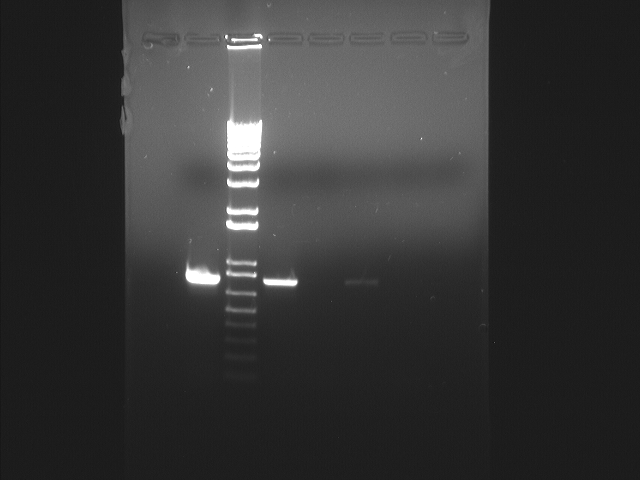


ACE (left side) and Atg (right side)


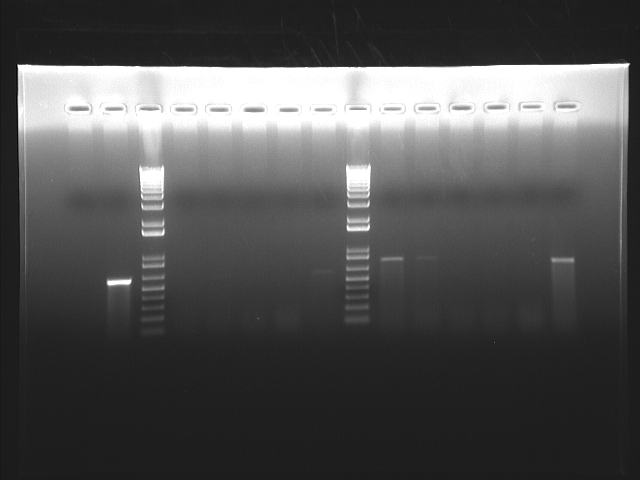


**Supplementary Video 1 Caption / Legend. Renin-bearing GFP^+^ B-1 lymphocytes interact with bacteria.**

GFP^+^ B-1 cells were incubated with CFP-expressing *E. Coli* bacteria. Time-lapsed pictures demonstrate that GFP^+^ B-1 lymphocytes interact with the bacteria via the assembly of pseudopod-like extensions.
